# The Non-Histone Protein FgNhp6 Is Involved in the Regulation of the Development, DON Biosynthesis, and Virulence of *Fusarium graminearum*

**DOI:** 10.3390/pathogens13070592

**Published:** 2024-07-16

**Authors:** Jiakuo Cao, Junbo Lv, Limin Zhang, Heng Li, Hao Ma, Yanxiang Zhao, Jinguang Huang

**Affiliations:** College of Plant Health and Medicine, Shandong Engineering Research Center for Environment-Friendly Agricultural Pest Management, Qingdao Agricultural University, Qingdao 266109, China; caojk@stu.qau.edu.cn (J.C.); jblyu@foxmail.com (J.L.); liheng@stu.qau.edu.cn (H.L.); mahao@stu.qau.edu.cn (H.M.)

**Keywords:** *Fusarium graminearum*, HMG protein, growth and development, mycotoxin, pathogenicity

## Abstract

*Fusarium graminearum* is the primary causative agent of Fusarium head blight (FHB), a devastating disease affecting cereals globally. The high-mobility group (HMG) of non-histone proteins constitutes vital architectural elements within chromatin, playing diverse roles in various biological processes in eukaryotic cells. Nonetheless, the specific functions of HMG proteins in *F. graminearum* have yet to be elucidated. Here, we identified 10 HMG proteins in *F. graminearum* and extensively characterized the biological roles of one HMGB protein, FgNhp6. We constructed the FgNhp6 deletion mutant and its complementary strains. With these strains, we confirmed the nuclear localization of FgNhp6 and discovered that the absence of FgNhp6 led to reduced radial growth accompanied by severe pigmentation defects, a significant reduction in conidial production, and a failure to produce perithecia. The ∆FgNhp6 mutant exhibited a markedly reduced pathogenicity on wheat coleoptiles and spikes, coupled with a significant increase in deoxynivalenol production. An RNA sequencing (RNA-seq) analysis indicated that FgNhp6 deletion influenced a wide array of metabolic pathways, particularly affecting several secondary metabolic pathways, such as sterol biosynthesis and aurofusarin biosynthesis. The findings of this study highlight the essential role of FgNhp6 in the regulation of the asexual and sexual reproduction, deoxynivalenol (DON) production, and pathogenicity of *F. graminearum*.

## 1. Introduction

*Fusarium graminearum* is the primary causal agent of Fusarium head blight (FHB), a devastating disease suffered by wheat and barley worldwide [1,2]. In addition to severe yield losses, *F. graminearum* produces mycotoxins such as deoxynivalenol (DON) and zearalenone (ZEA) in the infected grains, which pose serious threats to human and animal health [3]. At present, the use of fungicides in agricultural production is the main strategy for controlling the occurrence and damage of this disease. However, long-term and abusive use leads to drug resistance of the pathogen and a low efficacy against FHB [4]. A comprehensive understanding of the biological basis of *F. graminearum* will provide a foundation for the effective prevention and control of this disease.

High-mobility group (HMG) proteins are distinguished by their notably high electrophoretic mobility in polyacrylamide gels. They are chromosomal proteins and are categorized into three principal families according to their functional domains: HMGA, HMGB, and HMGN [5]. The HMGA family is characterized by a conserved DNA-binding motif known as the ‘AT-hook’, which preferentially associates with the minor groove of AT-rich sequences [6]. They usually bind to multiprotein complexes of transcription factors and cofactors. HMGN proteins feature a highly conserved nucleosomal binding domain (NBD) that specifically interacts with nucleosomes [7]. The hallmark of HMGB proteins is the HMG-box, typically comprising approximately 75 amino acid residues. This domain adopts a distinctive L-shaped structure, with the N-terminal strand and helix III forming the longer arm and helices I and II creating the shorter arm [6,8,9]. While most HMGB proteins contain two HMG-box domains, some possess only one, such as Nhp6A/B in *Saccharomyces cerevisiae*. Most HMGB proteins are DNA-binding proteins without sequence specificity, while some HMGBs function as specific transcription regulators (like Rox1 in *S*. *cerevisiae* [10]) and several play functions in both manners (like Irx1 in *S*. *cerevisiae* [11]).

In *S. cerevisiae*, there are seven identified HMGB proteins. Unlike their mammalian counterparts, most of these fungal proteins possess only a single HMG-box domain [12]. The functionally redundant paralogs Nhp6A and Nhp6B are among the first extensively studied HMGB proteins in fungi [13]. They play critical roles in DNA structuring, gene transcription, and chromatin modification. Similar to mammalian HMGB1, Nhp6A/B regulates the transcription of Polymerase II (Pol-II)-dependent genes through several mechanisms. It facilitates the formation of TBP/TFIIA/DNA complexes by modulating the interaction between TATA-binding protein (TBP) and promoter sites [14,15,16,17]. Additionally, its interaction with the yeast FACT complex underscores its role in transcription elongation by RNA Pol II through nucleosomal templates, which involves the removal of histone H2A-H2B dimers [14,18]. Moreover, Nhp6 is also involved in the transcription by Pol-III. For instance, the Pol-III-transcribed gene *SNR6* in vivo is decreased in ∆Nhp6AB mutants [19,20].

Genetic studies have revealed that the deletion of either Nhp6 gene did not result in any observable phenotypic changes, suggesting a level of functional redundancy [21]. However, the double mutant (∆Nhp6AB) exhibits slow growth, genomic instability, and a shortened lifespan phenotype, indicating the critical nature of these proteins in yeast viability [22]. In filamentous fungi, *Magnaporthe oryzae*, the HMGB protein Mnh6 is also indispensable for fungal growth and development, pathogenicity, and disease cycling [23]. Here, we reported the identification of HMGB proteins in another filamentous plant pathogen, *F. graminearum*, and characterized one of them, FgNhp6, the homolog of Nhp6B/Nhp6A in *S. cerevisiae*. The results demonstrated that FgNhp6 plays a crucial role in vegetative growth, asexual and sexual reproduction, DON biosynthesis, and virulence, which also suggested the functional conservation of the Nhp6 protein.

## 2. Materials and Methods

### 2.1. Strains and Culture Conditions

The *F. graminearum* strain PH-1 (NRRL 31084) was used as the parental strain for target gene deletion in this study. All *F. graminearum* strains were cultured on potato dextrose agar (PDA) or in potato dextrose broth (PDB) at 25 °C, unless otherwise stated. All strains were maintained as conidial suspensions for long-term storage in 15% glycerol at −80 °C.

### 2.2. Protein Identification and Sequence Analysis

To identify all the HMG proteins in *F. graminearum*, the hidden Markov model specific to the HMG box domain (PF00505) was employed in an HMMER search [24] against the whole protein dataset deduced from the *F. graminearum* reference genome (GCF_000240135.3, accessed on 20 May 2022). Hits with an e-value of ≤1 × 10^−5^ were considered to be the putative HMG proteins and were further validated by the Simple Modular Architecture Research Tool (SMART) [25]. To extend our study, we performed a homology search using the FgNhp6 amino acid sequence as a query via the NCBI Genbank database using the Basic Local Alignment Search Tool (BLAST) (v2.12.0). Then, we retrieved the complete amino acid sequences of Nhp6 homologs from several species. The MAFFT program (v7.496) [26] was engaged for the sequence alignment, and the result was visualized using TBtools-II [27]. Subsequently, a phylogenetic tree was constructed using the maximum likelihood method with the MEGA-X software (v10.2.6) [28] to clarify the evolutionary relationships among the Nhp6 homologs.

The GenBank accession numbers for the analyzed sequences are listed as follows: *Fusarium graminearum* (XP_011316047.1), *Saccharomyces cerevisiae* (Nhp6B, NP_009647.2; Nhp6A, NP_015377.1), *Pyricularia oryzae* (XP_003710967.1), *F. verticillioides* (XP_018743258.1), *F. fujikuroi* (XP_023424625.1), *Botrytis cinerea* (XP_024551858.1), *Neurospora crassa* (XP_011393295.1), *Aspergillus fumigatus* (XP_754458.1), *A. nidulans* (XP_660489.1), *Ustilaginoidea virens* (XP_042995499.1), and *Penicillium expansum* (XP_016594384.1).

### 2.3. Construction of FgNhp6 Deletion Mutants and Complementary Strains

To obtain the FgNhp6 deletion mutant (∆FgNhp6), the *FgNhp6* gene was replaced with the hygromycin B phosphotransferase gene (*HPH*) using the split-marker method (Figure S2) [29]. First, the upstream and downstream flanking sequences of *FgNhp6* were amplified from *F. graminearum* genomic DNA. Two fragments of the *HPH* gene were amplified from the pKH plasmid. The resulting PCR products were fused and amplified for transformation, as described before [30]. The obtained hygromycin-resistant transformants were first identified by PCR and further verified by Southern blotting using the DIG High Prime DNA Labeling and Detection Starter Kit I (Roche Diagnostics, Mannheim, Germany), following the manufacturer’s instructions. For complementation assay, a 3.0 kb fragment consisting of the *FgNhp6* gene with about 1.4 kb of upstream sequence and about 0.3 kb of downstream sequence was amplified from *F. graminearum* genomic DNA and then subcloned into the pKN vector. The resulting pKN-FgNhp6 plasmid was then introduced into the ∆FgNhp6 mutant using the PEG-mediated protoplast transformation method, yielding the complementary strain ∆FgNhp6-C. All the primers used in this study are listed in Table S4.

### 2.4. Subcellular Localization of FgNhp6

To elucidate the subcellular distribution of FgNhp6, a strain expressing GFP-tagged FgNhp6 at the C-terminal was constructed using a similar method to the construction of the complementary strain. Specifically, a DNA fragment encompassing the *FgNhp6* gene, under the control of its native promoter and devoid of the stop codon, was cloned into the pRGTN vector digested with *Kpn*I and *Bam*HI. The resultant recombinant plasmid was subsequently introduced into the ∆FgNhp6 mutant strain to generate the ∆FgNhp6::FgNhp6-GFP strain. The fresh hypha of this strain was examined using an Axio Scope A1 fluorescence microscope (Zeiss, Jena, Germany). To observe the locations of nuclei, hyphae were stained with 10 g/mL 4′-6-diamidino-2-phenylindole (DAPI) 10 min prior to microscopic examinations.

### 2.5. Evaluation of Conidial Production and Sexual Development

Five mycelium plugs (5 mm in diameter) were excised from the periphery of 4-day-old colonies of each fungal strain. These plugs were then incubated in an orbital shaker at 200 rpm for 5 days at 25 °C in carboxymethyl cellulose (CMC) medium (1.5 g of carboxymethyl cellulose, 0.2 g of NaNO_3_, 0.05 g of MgSO_4_∙7H_2_O, 0.1 g of KH_2_PO_4_, and 0.1 g of yeast extract in 100 mL of distilled water). Subsequently, the number of conidia was measured utilizing a hemocytometer. For the assessment of sexual reproduction, each strain was grown on carrot agar plates (200 g of crushed carrot and 9 g of agar in 1 L of distilled water) for 7 days. Then aerial hyphae were scraped off from the surface of the plates, and 1 mL of sterile 2.5% Tween 60 solution was added to every plate. The plates were then incubated for an additional 2–3 weeks at 25 °C under black light. The removal of aerial hyphae and the addition of Tween 60 solution were replicated whenever the aerial hyphae reappeared during this incubation period. After that, perithecium formation was examined. This experiment was repeated three times.

### 2.6. Pathogenicity Assays

For pathogenicity assays, the FHB-susceptible wheat cultivar “Jimai22” was employed to assess the virulence of each *F. graminearum* strain on wheat coleoptiles and wheat heads. First, conidia of each strain were prepared in a CMC medium, as described above. The conidia were harvested by centrifugation, resuspended in sterile distilled water, and adjusted to a concentration of 1 × 10^6^ spores/mL. In the coleoptile infection assay, 10 μL of the prepared spore suspension was applied to the wheat coleoptiles, with sterile distilled water serving as the negative control. The length of the lesions on the coleoptiles was recorded at 10 days post-inoculation. At least 10 wheat coleoptiles were inoculated with each strain, and this assay was performed in triplicate. In the wheat head infection assay, during the wheat mid-flowering stage, 10 L of spore suspension was carefully inoculated into the floret in the fifth spikelet from the bottom of the wheat head. Wheat heads inoculated with sterile distilled water were used as a control. After 14 days, the infected spikelets in each wheat head were counted and used to evaluate the virulence of each strain. Similar to the coleoptile assay, at least 10 wheat heads were inoculated with each strain, and this experiment was carried out three times independently.

### 2.7. Assessment of DON Production

First, 1 × 10^6^ conidia of each strain were prepared as mentioned above and then transferred into trichothecene biosynthesis induction (TBI) medium, where 30 g of sucrose, 800 mg of putrescine, 1 g of KH_2_PO_4_, 0.5 g of MgSO_4_·7H_2_O, 0.5 g of KCl, 10 mg of FeSO_4_·7H_2_O, and 200 μL of trace element solution and ddH_2_O were added to 1 L and autoclaved. The trace element solution was made up of 5 g of citric acid, 5 g of ZnSO_4_·7H_2_O, 0.25 g of CuSO_4_·5H_2_O, 50 mg of MnSO_4_·H_2_O, 50 mg of H_3_BO_3_, and 50 mg of NaMoO_4_·2H_2_O in 100 mL of ddH_2_O [31]. After incubation for seven days, a competitive indirect ELISA experiment using the filtrate of the culture suspension was performed to determine the TBI production. The experiments were conducted with a DON detection kit (Huanan Magtech Bio-Tech, Beijing, China), according to the manufacturer’s instructions. The mycelia in the culture suspension were collected, dried, and weighed for calculation. This experiment was repeated three times independently, and each time, with five replicates for each strain.

### 2.8. RNA Isolation and RNA-Sequencing Analysis

The mycelia of *F. graminearum* wild-type strain PH-1 and deletion mutant ∆FgNhp6 were collected from the PDB cultures after incubation for 2 days. For RNA-seq, three replicates were prepared for each strain. The total RNA of each sample was extracted using Trizol reagent (Thermo Fisher, Waltham, MA, USA) following the manufacturer’s procedure. Subsequently, RNA libraries were constructed and sequenced on the Illumina Novaseq^TM^ 6000 platform by LC Bio-Technology CO., Ltd. (Hangzhou, China). A total of 2 million 150 bp paired-end reads were generated using the Illumina paired-end RNA-seq approach. To obtain high-quality data, the reads were then filtered by Cutadapt (v1.9). The raw sequence data were submitted to the NCBI Short Read Archive (SRA) with the accession number PRJNA1100473. For further analysis, the cleaned reads of all samples were aligned to the *F. graminearum* reference genome (GCF_000240135.3) using the HISAT2 package (v2.2.1) [32]. DESeq2 software (v1.22.2) [33] was used to identify differentially expressed genes (DEGs). Genes with the parameter of false discovery rate (FDR) below 0.05 and an absolute fold change of ≥2 were considered to be DEGs. The DEGs were then subjected to an enrichment analysis of Gene Ontology (GO) functions [34] and Kyoto Encyclopedia of Genes and Genomes (KEGG) pathways [35]. In the enrichment analysis, a term or a pathway with a *p*-value of <0.05 was considered as significant.

### 2.9. qRT-PCR Assays

Mycelia were prepared for RNA extraction as mentioned above. Total RNA was extracted using the SteadyPure Universal RNA Extraction Kit (Accurate Biotech, Changsha, China). Reverse transcription was performed using HiScriptII Q RT SuperMix for qPCR (+gDNA wiper) (Vazyme Biotech, Nanjing, China). The relative expression level of each gene was determined using ChamQ SYBR qPCR Master Mix (Vazyme Biotech, Nanjing, China), with the primers listed in Table S4. All the operations strictly followed the instructions of these kits. The relative expression level of the target gene was calculated using the 2^−∆∆Cq^ method with the β-actin gene (FGSG_07335) as the internal control [36]. This experiment was conducted three times independently, each with three replicates.

### 2.10. Statistical Analyses

In this study, SPSS software (v19.0) was employed to perform the statistical analysis of the experimental data, except the RNA-seq data. The mean values from triplicate experiments were analyzed using ANOVA to detect significant differences. Duncan’s multiple range test was applied for post hoc multiple comparisons upon identifying significant variance. Data are reported as the mean value ± standard errors.

## 3. Results

### 3.1. Identification of the HMG-box Containing Proteins in F. graminearum

**Figure 1:**
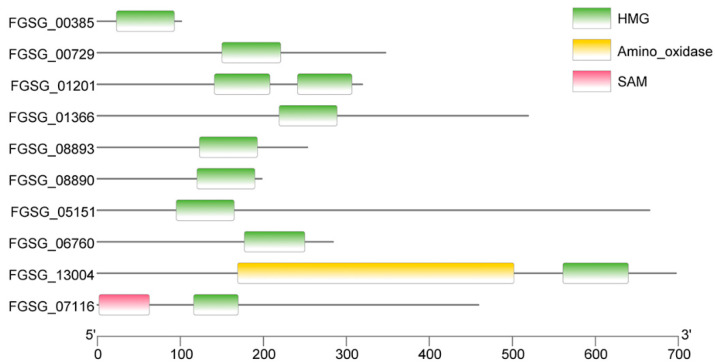
Illustration of domain architecture. Protein domains of each HMG protein in *F. graminearum* are shown. These domains were identified by the SMART server.

In order to identify the HMG proteins in *F. graminearum*, we conducted an HMMER search using the hidden Markov model of the HMG box domain (PF00505) against all the proteins derived from the *F. graminearum* reference genome (GCF_000240135.3, accessed on 20 May 2022). Our analysis revealed a total of 10 HMG domain-containing proteins, with a significance threshold (e-value) of ≤1 × 10^−5^. The putative HMG domain-containing proteins exhibited a range of sizes, with the smallest protein (FGSG_00385) consisting of 101 amino acid residues and the largest protein (FGSG_13004) consisting of 697 residues. However, it is important to note that, except for the HMG-box domain, only limited domains within these proteins were identified by the SMART server (Figure 1).

In the previous study, we explored the reshaping of lysine 2-hydroxyisobutyrylome in *F. graminearum* by tebuconazole treatment and found that, among 10 HMG-box containing proteins, changes in lysine 2-hydroxyisobutyrylation (Khib) were observed in only two proteins. Specifically, the modification levels of two lysine residues in FGSG_00385 were notably increased, while the modification level of a single lysine residue in FGSG_01201 was significantly reduced [35]. In this study, we then directed our attention to the gene *FGSG_00385* to reveal its functional roles, especially its roles in fungicide sensitivity. This gene is composed of 1303 base pairs and consists of four exons, with a large 5′ untranslated region (5′UTR) and a large 3′ untranslated region (3′UTR). A homologous analysis indicated that the protein encoded by FGSG_00385 shared a high sequence identity with yeast non-histone proteins Nhp6B and Nhp6A. It exhibited a 72.84% amino acid sequence identity and 80% sequence coverage with Nhp6B, as well as a 74.32% sequence identity and 73% coverage with Nhp6A. So, hereafter, we designate it as FgNhp6. Multiple sequence alignment has shown that the FgNhp6 protein is highly conserved evolutionarily (Figure S1).

### 3.2. FgNhp6 Localizes to the Nucleus

To further characterize the function of FgNhp6, the deletion mutants ∆FgNhp6-1 and ∆FgNhp6-2 were constructed via a homologous recombination strategy and confirmed by Southern blotting (Figure S2). Subsequently, the *FgNhp6* gene with its native promoter and *FgNhp6-GFP* fusion gene with the strong constitutive promoter RP27 were reintroduced into one of the deletion mutants to create the complementary strains ∆FgNhp6-C and ∆FgNhp6::FgNhp6-GFP, respectively.

**Figure 2:**
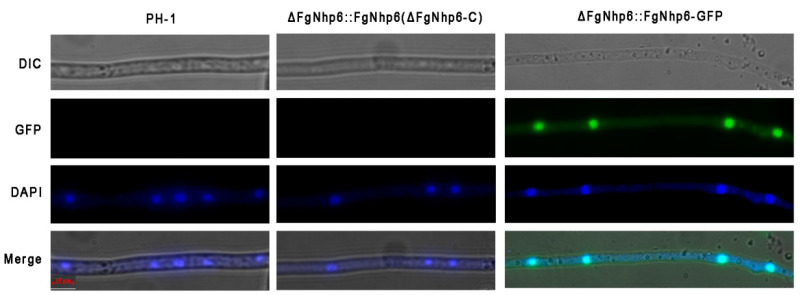
Subcellular localization of FgNhp6 in *F. graminearum*. The subcellular localization of the GFP fusion protein in ΔFgNhp6::FgNhp6-GFP strain was observed by fluorescence microscopy. Nuclei were first stained with DAPI. Bar = 10 μm.

As the putative homolog of the non-histone protein Nhp6, it is postulated that FgNhp6 primarily resides within the nucleus. We first confirmed this by microscopic examination using the ∆FgNhp6::FgNhp6-GFP strain. Fluorescence microscopy revealed a substantial colocalization between the green fluorescent protein (GFP) signal and the nuclear stain 4′-6-diamidino-2-phenylindole (DAPI) (Figure 2). This colocalization indicated that FgNhp6-GFP was predominantly localized to the nucleus.

### 3.3. FgNhp6 Is Involved in Vegetative Growth and Pigmentation in F. graminearum

**Figure 3:**
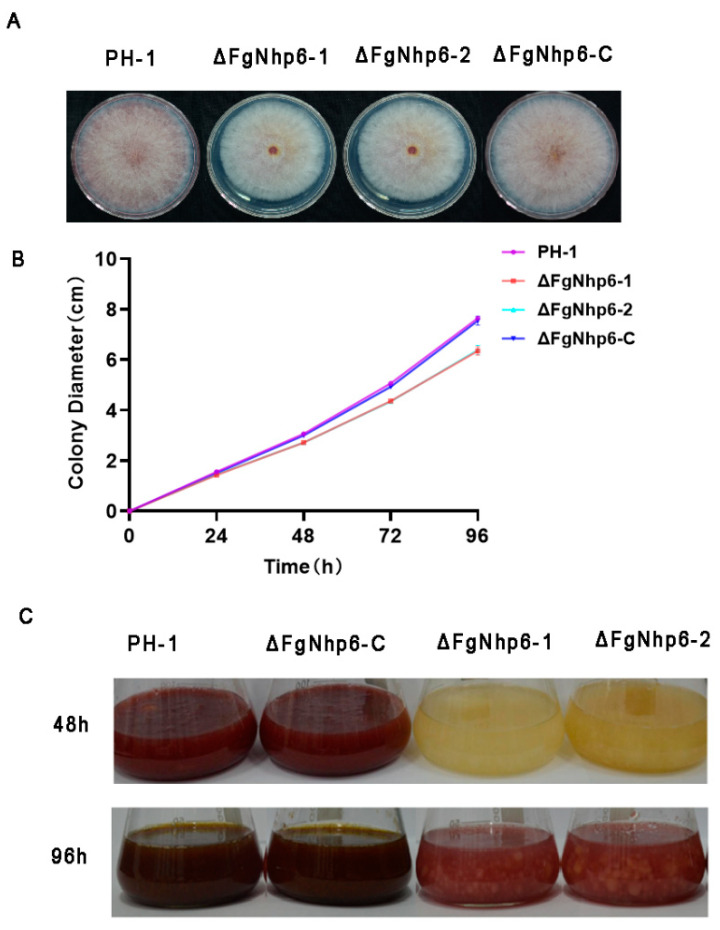
FgNhp6 is involved in the regulation of vegetative growth and pigmentation in *F. graminearum.* (**A**) Colony morphology of the wild-type strain PH-1, the ΔFgNhp6 mutants, and the complementary ΔFgNhp6-C strain. Photos were taken after strains were incubated on PDA plates at 25 °C for 4 days. (**B**) The radial growth rate on PDA plates of these strains, which were cultured on PDA plates for 4 days at 25 °C, and the colony diameters of each strain were measured every 24 h. The experiments were repeated three times, with three replicates each time. (**C**) The pigmentation of the above strains. The photograph was taken at 48 h or 96 h after the strains were incubated in the PDB medium.

We then assessed the colony morphology and vegetative growth of these strains on PDA (potato dextrose agar) medium. Following a 96 h incubation period at 25 °C, the wild-type strain PH-1 and the complementary strain ∆FgNhp6-C exhibited average colony diameters of 7.65 ± 0.04 cm and 7.55 ± 0.15 cm, respectively. However, the deletion mutants ∆FgNhp6-1 and ∆FgNhp6-2 presented significantly reduced diameters, measuring only 6.35 ± 0.14 cm and 6.38 ± 0.15 cm, respectively (Figure 3A,B). These findings suggest that the ablation of the FgNhp6 gene negatively affected the vegetative growth capacity of *F. graminearum* on PDA media.

Additionally, notable differences were observed in the pigmentation of the colonies: the deletion mutants ∆FgNhp6-1 and ∆FgNhp6-2 exhibited a consistently paler hue in comparison to the wild-type strain PH-1 and the complementary strain ∆FgNhp6-C (Figure 3A). This disparity in coloration became even more pronounced when the strains were cultured in PDB liquid medium for 48 h, and persisted after 96 h of growth, with the deletion mutants maintaining a lighter pigmentation (Figure 3C). This phenotypic change suggested a possible reduction in the synthesis of the pigment aurofusarin in the absence of FgNhp6. Collectively, our observations affirm that FgNhp6 plays a critical role in the regulation of vegetative growth and aurofusarin biosynthesis in *F. graminearum*.

### 3.4. FgNhp6 Played a Critical Role in Both Asexual and Sexual Developmental Phases

**Figure 4:**
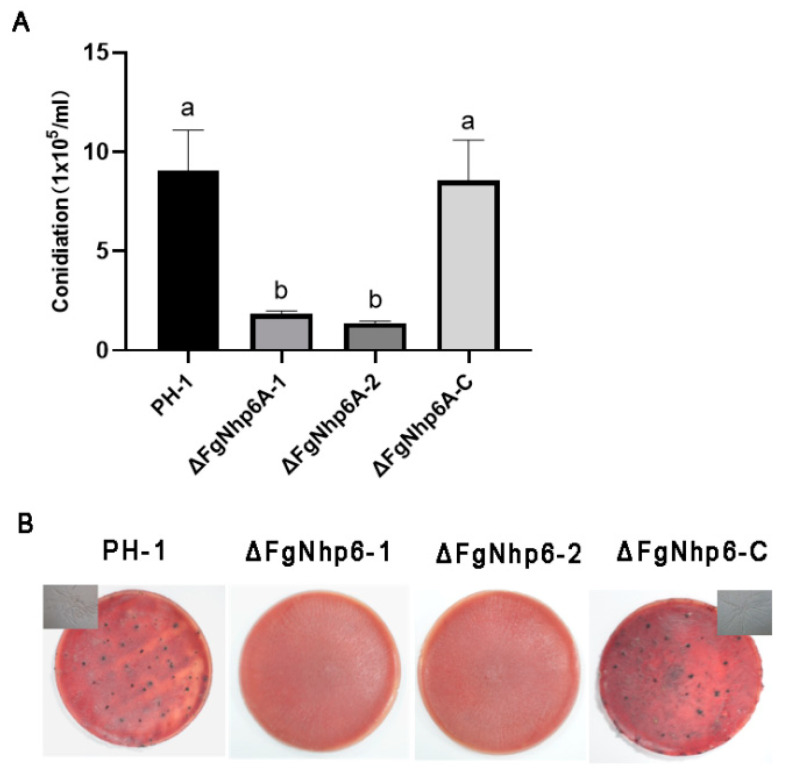
FgNhp6 plays roles in asexual and sexual reproduction. (**A**) Conidiation of each strain. Conidia production was determined after each strain was incubated in CMC medium at 25 °C and 200 rpm for 4 days. (**B**) Perithecia and ascospore formation. Strains were first cultured on carrot agar for 7 d and then mock-fertilized to induce sexual reproduction using a Tween-60 solution. The formation of perithecia was observed 3 weeks after an additional incubation. The ascospores of PH-1 and FgNhp6-C strains were shown on the corner. All experiments were performed in triplicate, with three replicates each time. The standard errors of the three experiments are represented by error bars, with different letters above the bars marking a statistically significant difference at the *p* < 0.05 level.

We explored the potential involvement of FgNhp6 in both the asexual and sexual reproductive processes of different *F. graminearum* strains: PH-1, ∆FgNhp6-1, ∆FgNhp6-2, and ∆FgNhp6-C. First, we performed an assessment of conidial output in the CMC medium. The results indicated a significant reduction in conidial generation resulting from the disruptions of the ΔFgNhp6 gene. Specifically, after a 5-day incubation period, the ∆FgNhp6-1 and ∆FgNhp6-2 mutants produced markedly lower concentrations of macroconidia, measuring 1.83 ± 0.67 × 10^5^ and 1.39 ± 0.46 × 10^5^ macroconidia/mL, respectively. In contrast, the PH-1 and ∆FgNhp6-C strains exhibited higher macroconidia production, with counts of 9.06 ± 2.94 × 10^5^ and 8.61 ± 2.90 × 10^5^ macroconidia/mL, respectively (Figure 4A). Furthermore, the assessment of sexual reproduction via perithecia formation on the CA plates revealed stark contrasts. The PH-1 and ∆FgNhp6-C strains successfully produced normal perithecia, asci, and ascospores, indicative of unimpaired sexual reproduction. Conversely, such structures were conspicuously absent in the deletion mutants, underlining the inhibition of sexual reproduction upon FgNhp6 deletion (Figure 4B). Therefore, our findings demonstrated that FgNhp6 was essential for both the asexual and sexual development of *F. graminearum*.

### 3.5. ΔFgNhp6 Is Indispensable for Full Virulence in F. graminearum

**Figure 5:**
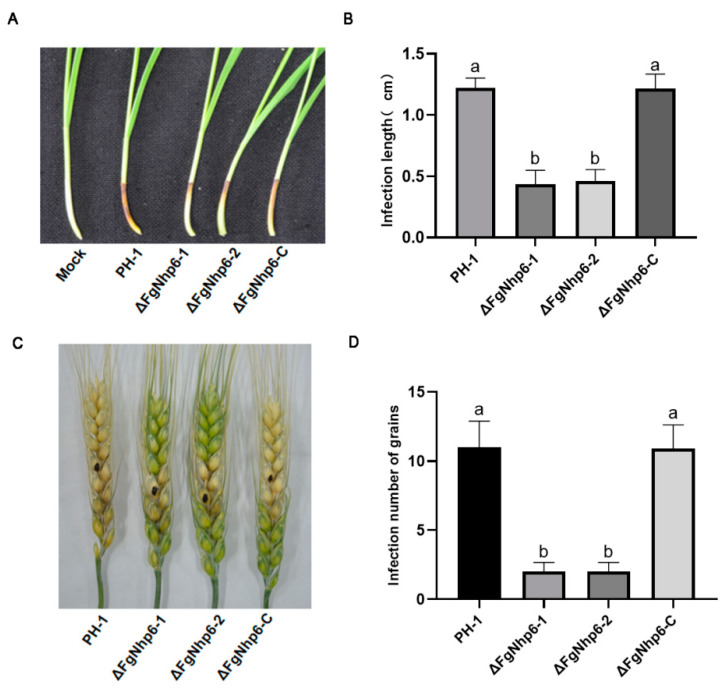
FgNhp6 is required for full virulence of *Fusarium graminearum*. (**A**) The representative wheat coleoptiles infected by *F. graminearum* PH-1 and ΔFgNhp6 mutants. (**B**) Lesion length of wheat coleoptiles infected by *F. graminearum* PH-1 and ΔFgNhp6 mutants. (**C**) The representative wheat heads infected by *F. graminearum* PH-1 and ΔFgNhp6 mutants. (**D**) The number of grains infected by *F. graminearum* PH-1 and ΔFgNhp6 mutants. Error bars showed the standard deviation. The different letters indicate statistically significant differences at the *p* < 0.05 level.

Given the significance of *F. graminearum* as a critical plant pathogen, the involvement of ΔFgNhp6 in its pathogenicity was also investigated. The virulence of *F. graminearum* strains PH-1, ∆FgNhp6-1, ∆FgNhp6-2, and ∆FgNhp6-C was tested on both flowering wheat heads and wheat coleoptiles. In the wheat coleoptile assay, the lesion lengths of the infected coleoptiles were measured. Our data indicated that the wild-type PH-1 and the complementary ∆FgNhp6-C strains elicited extensive infection areas, with mean lesion lengths of 4.00 ± 0.08 cm and 3.80 ± 0.16 cm, respectively. Contrastingly, the deletion mutants ∆FgNhp6-1 and ∆FgNhp6-2 exhibited significantly reduced lesion lengths of 1.10 ± 0.16 cm and 1.13 ± 0.12 cm, respectively (Figure 5A,B). Similar findings were also observed in the virulence assays on flowering wheat heads. The ∆FgNhp6 mutants were confined to infecting only 5–6 grains proximal to the inoculation point, whereas the wild-type strain exhibited a wider range of infection, impacting 7–13 grains (Figure 5C,D). This contrast highlighted the deletion of FgNhp6 as a substantial detriment to *F. graminearum* virulence. These findings suggested that FgNhp6 was crucial for full virulence in *F. graminearum*.

### 3.6. FgNhp6 Negatively Regulated the DON Biosynthesis

**Figure 6:**
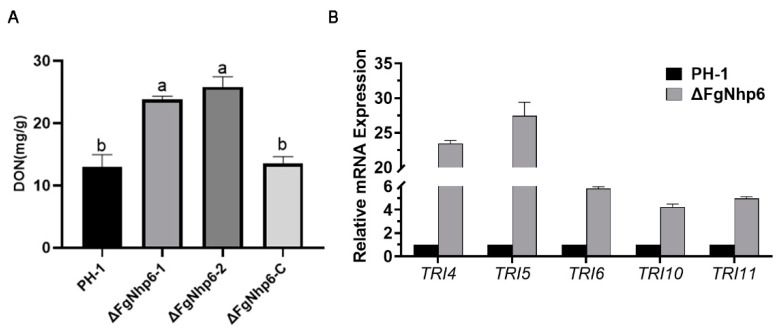
FgNhp6 negatively regulates the deoxynivalenol (DON) biosynthesis in *F. graminearum*. (**A**) DON production level of each strain. DON production was measured per gram of dried mycelia after strains were cultured in TBI medium for 7 days. (**B**) Relative mRNA levels of several *TRI* genes in wild-type strain and deletion mutant. The relative expression levels were calculated with 2^−ΔΔCq^ method using the actin gene as the internal control, and the expression of each gene in wild type strain was set to 1. Standard errors of three independent experiments were shown on the bar. Different letters indicate significantly different at the *p* < 0.05 level.

DON is a primary mycotoxin and a crucial virulence factor in *F. graminearum*. To assess the effect of FgNhp6 deletion on the DON-producing capacity and explore the reasons that led to the reduced pathogenicity of the FgNhp6 deletion mutants, we determined the DON production in each strain. This was performed using an ELISA kit after the *F. graminearum* strains were cultured in TBI medium to induce DON biosynthesis. Notably, after a seven-day incubation in the TBI medium, DON production was significantly increased in the FgNhp6 deletion mutants. The ∆FgNhp6-1 and ∆FgNhp6-2 strains yielded 24.15 ± 0.65 mg and 26.03 ± 1.11 mg of toxin per gram of dry mycelium, respectively. This was in stark contrast to the DON levels of 12.93 ± 1.65 mg and 13.50 ± 0.80 mg produced by the PH-1 and ΔFgNhp6-C strains, respectively (Figure 6A). These findings were statistically validated to affirm the elevated DON production in the ∆FgNhp6 mutants as significant (*p* < 0.05), implying a negative regulatory function of the *FgNhp6* gene in the biosynthesis of DON by *F. graminearum*. To further confirm this finding, we used qPCR to detect the relative transcriptional expression levels of several genes essential for DON biosynthesis. In the ∆FgNhp6 mutants, a significant upregulation in the expression of *TRI4*, *TRI5*, *TRI6*, *TRI10*, and *TRI11* was observed compared with the wild-type PH-1 strain (Figure 6B). This elevation indicated that the FgNhp6 gene exerted control over the DON biosynthetic pathway by modulating the *TRI* gene cluster.

### 3.7. RNA Seq Analysis with ∆FgNhp6 Mutant

**Figure 7:**
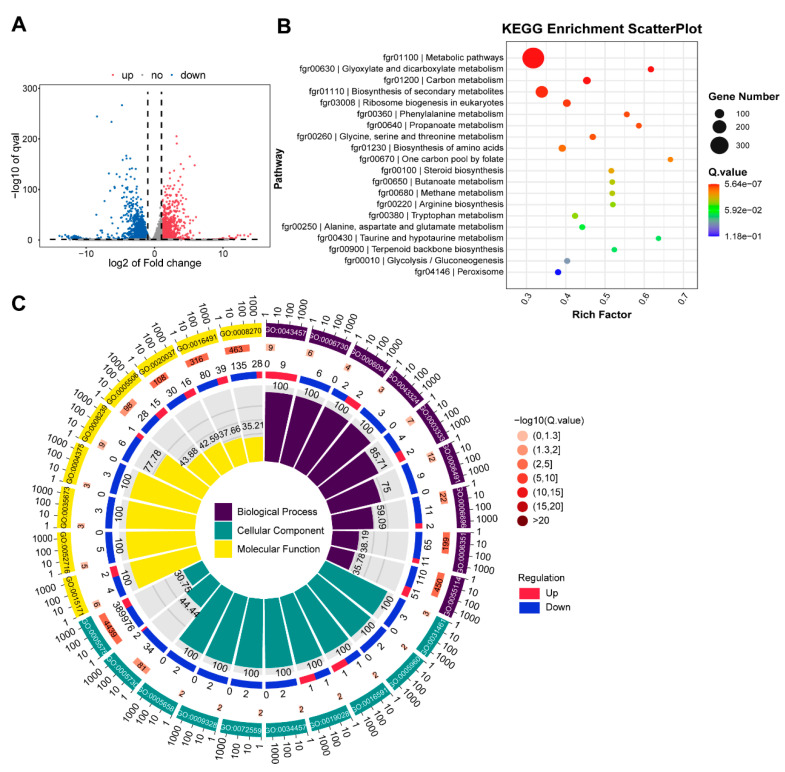
RNA-sequencing analysis of *Fusarium graminearum* wild-type strain PH-1 and FgNhp6 deletion mutant strain. (**A**) Volcano plot of all identified genes. The differentially expressed genes (*padj* < 0.05 and |log2 of fold change| > 1) are shown in blue dots (downregulated) or red dots (upregulated). (**B**) A LoopCircos plot of the result of GO enrichment analysis. The first ring (from the outermost to the innermost) represents the top-enriched GO terms based on the smallest Q-values; the second ring represents the number of genes annotated to GO terms, with colors depicting the -log10 values of the enrichment analysis Q-values; the third ring shows the number of DEGs in each GO term; the fourth ring displays the percentage of the enrichment factor. (**C**) A scatter plot of the result of KEGG enrichment analysis.

To identify the genes regulated by FgNhp6, RNA-seq was performed with RNA isolated from the *F. graminearum* PH-1 and ΔFgNhp6 strains grown in PDB medium for two days. Applying a threshold of fold changes greater than two, a total of 3672 differentially expressed genes (DEGs) were identified. Compared with the PH-1 strain, 1046 genes were upregulated and 2626 genes were downregulated in the FgNhp6 deletion mutant strain (Figure 7A, Table S1). A GO enrichment analysis of the DEGs revealed that the enriched GO terms were predominantly associated with downregulated genes. The most significant terms within the cellular component, biological process, and molecular function groups were cellular component (GO:0005575), obsolete-oxidation-reduction process (GO: 0055114), and zinc ion binding (GO:0008270), respectively (Figure 7B, Table S2). In KEGG enrichment, fifteen KEGG pathways were significantly enriched. The three most significant pathways included metabolic pathways (fgr01100), glyoxylate and dicarboxylate metabolism (fgr00630), and carbon metabolism (fgr01200). Notably, the pathway “biosynthesis of secondary metabolites” (fgr01110) was also significantly enriched (Figure 7C, Table S3). Among these DEGs, many genes were believed to be involved in the growth and development of *F. graminearum* or associated with its virulence (Table 1); most of them were downregulated. For example, several genes in the sterol synthesis and aurofusarin biosynthesis pathway were downregulated. The former is essential for the fungal membrane structure, and the latter may be responsible for delayed pigmentation. Furthermore, many genes involved in pathogenicity were also downregulated, such as *FgPex1* and *Chs1* [37,38].

## 4. Discussion

HMGB proteins are multifunctional, playing crucial roles in DNA repair, transcription regulation, and chromatin modifications [48,49,50]. However, only a limited number of HMGB homologs in fungi have been characterized to date. In *S. cerevisiae*, seven HMGB proteins have been identified and characterized [22]. Similarly, several homologs in other yeasts have also been reported, including YlMhb1 from *Yarrowia lipolytica*, Gcf1 from *Candida parapsilosis*, and KlRox1 from *Kluyveromyces latis* [51,52,53]. In filamentous fungi, 12 HMG-box proteins were identified and functionally characterized from the ascomycete *Podospora anserina* [54]. Additionally, seven HMGB proteins were detected in *Aspergillus nidulans*, with HmbA, HmbB, and HmbC being extensively studied [55,56,57]. For plant pathogens, Mnh6 from *Magnaporthe oryzae* has been reported. Recently, 10 HMG-box-containing proteins were identified in *Fusarium fujikuroi*, with FfHmbC undergoing deep investigation [58]. In this study, we reported on the HMGB proteins in another well-known pathogen, *Fusarium graminearum*, where 10 HMGB proteins were identified. Furthermore, we conducted an in-depth exploration of the roles of FgNhp6.

FgNhp6 is a small protein consisting of 101 amino acid residues. A SMART search identified an HMG-box domain located at amino acid positions 23–93. A homology analysis indicated that FgNhp6 is a homolog of yeast Nhp6B/Nhp6A. Furthermore, sequence alignment revealed a high level of sequence identity among the Nhp6 homologs, while a phylogenetic analysis further demonstrated that the Nhp6 protein is highly evolutionary conserved.

In yeast, no phenotypic change can be observed with the deletion of either Nhp6 protein. However, the double mutant showed slow growth [21]. In *F. graminearum*, there was only one homolog of Nhp6. Consistent with our expectations, the deletion of FgNhp6 also led to slow radial growth. Furthermore, the biosynthesis of aurofusarin was notably delayed, attributable to the decreased expression of the essential biosynthesis genes. Sexual and asexual reproduction are essential processes of the fungal life cycle and play major roles in primary infection and secondary infection, respectively [2]. Conidia is the main secondary inoculum source, and our findings indicated that the deletion of FgNhp6 would decrease conidia production and block perithecium formation. This means that FgNhp6 is also of great importance for the occurrence and spread of FHB disease in the field.

As a predominant pathogen of FHB, we assessed the pathogenicity of both *F. graminearum* wild-type and the ΔFgNhp6 mutant on wheat coleoptiles and wheat heads. Our results indicated that the deletion of FgNhp6 led to a significant decrease in *F. graminearum* virulence. DON, a mycotoxin produced by some *Fusarium* species, is both phytotoxic and toxic to animals [59,60]. It can inhibit protein synthesis and induce apoptosis by binding to the 60S ribosomal subunit of eukaryotic cells [61]. DON is considered to be a critical virulence factor of *F. graminearum* [62]. It can promote the spreading of pathogens from infected florets to neighboring ones through the rachis [63]. Thus, an ELISA assay was conducted to evaluate the capacity of DON production in TBI medium [31,64], which contains putrescine, potentially playing a role in DON induction in the infected wheat spikes [65]. However, contrary to our expectations, the results revealed that DON production was significantly elevated, suggesting that FgNhp6 may negatively regulate DON biosynthesis. Moreover, a qPCR analysis of the essential genes involved in DON biosynthesis demonstrated a significant increase in the expression levels of all tested genes, consistent with the increased levels of DON. Previous studies have observed a reduced virulence coupled with an enhanced DON production in the deletion mutants of genes like *FgSah1*, *Pmr1*, *FgGdt1*, *FgGyp1*, *FgNot2*, and *FgNot3* [66,67,68,69]. Our study showed slower vegetative growth in the ΔFgNhp6 mutant, suggesting it might struggle with hyphal growth within plant tissues, which may contribute to its reduced pathogenicity. The ΔFgNhp6 mutant may also exhibit impaired penetration during the initial stages of infection, similar to FgSah1 deletion mutants [68]. Meanwhile, RNA-seq analysis indicated a decrease in the expression of many genes associated with pathogenicity in the FgNhp6 deletion mutant strain, such as genes associated with peroxisomes and chitinase genes, which may also contribute to reduced pathogenicity. The in-depth mechanisms behind reductions in pathogenicity require further investigation.

The RNA-seq data indicated that the expressions of thousands of genes were affected due to the absence of FgNhp6. These DEGs were mainly enriched in several metabolism pathways, which is consistent with the fundamental biological roles of FgNhp6. Significantly, the secondary metabolism pathway was especially enriched, which can be favored by the phenotypic changes we found: delayed pigmentation and increased DON production.

Collectively, the deletion of FgNhp6 led to a significant reduction in vegetative growth, pigmentation, conidiation, and pathogenicity, while also blocking sexual reproduction and enhancing DON production. In *P. anserina*, the deletion mutant of PaHMG6, the homolog of Nhp6, exhibits low female fertility and produces only a few perithecia. The ΔPaHMG6 strain also showed a decrease in growth [54]. In *A. nidulans*, the Nhp6 homolog HmbA is also required for normal growth, ascospore production, and viability [56]. In the plant pathogen *M. oryzae*, the loss of Mnh6 will lead to significantly reduced vegetative growth and conidiation. The pathogenicity of ΔMnh6 is significantly reduced [23]. Taken together, the function of Nhp6 proteins in vegetable growth and development is conserved among different species.

2-hydroxyisobutyrylation adds a 2-hydroxyisobutyryl group to the lysine residue, which abolishes the positive charge and introduces a hydroxyl group. This modification can be observed in both histone and non-histone proteins, and has been found to be involved in diverse biological processes, like the regulation of gene expression and secondary metabolism [70]. In our previous study, we discovered that FgNhp6 is 2-hydroxyisobutyrylated, and the modification abundance was affected by tebuconazole. In this study, we tried to determine the EC_50_ value of tebuconazole to *F. graminearum* wild-type strain and the FgNhp6 deletion mutant. However, we did not observe any significant changes. Nevertheless, both RNA-seq data and qPCR analysis revealed a significant decrease in the relative expression levels of three *CYP51* genes, which are the target coding genes. The underlying mechanism of this still requires further investigation.

## 5. Conclusions

In conclusion, the deletion of FgNhp6 resulted in pleiotropic effects, highlighting its critical role in the regulation of both asexual and sexual reproduction, DON synthesis, and the pathogenicity of *F. graminearum*. While more investigations are needed to elucidate these intricate molecular mechanisms, this research offers highly valuable insights to enhance our comprehensive understanding of FgNhp6′s role in *F. graminearum*.

## Figures and Tables

**Table 1:** Several DEGs that may be associated with the phenotypic changes.

Protein Name	Gene Locus	Log2FC (RNA-seq)	Log2FC (qPCR)	Reference
Sterol-biosynthesis-related genes				
FgCYP51A	FGSG_04092	−6.30	−4.54	[39]
FgCYP51B	FGSG_01000	−1.69	−1.43	[39]
FgERG9	FGSG_09381	−2.76	−2.87	[40]
FgCYP51C	FGSG_11024	−0.48	−0.48	[39]
Aurofusarin biosynthesis gene cluster				
GIP1	FGSG_02328	−3.36	−3.06	[41]
AurJ	FGSG_02326	−3.34	−2.90	[41]
AurF	FGSG_02327	−3.36	−3.49	[41]
PKS12	FGSG_02324	−4.18	−4.45	[41]
Transporters				
	FGSG_08308	−2.11	−2.15	[42]
	FGSG_08309	−2.79	−2.61	[42]
	FGSG_08749	−2.42	−3.10	[43]
	FGSG_03882	5.85	5.00	[42]
	FGSG_05096	2.21	1.72	[44]
Other genes related to virulence				
FgPex1	FGSG_07104	−1.75	−2.11	[37]
FgCrz1A	FGSG_13711	−1.65	−1.30	[45]
FgChs1	FGSG_10327	−1.77	−1.71	[38]
FgChs2	FGSG_02483	−1.53	−1.74	[46]
FgSIRT2	FGSG_09218	−1.40	−1.42	[47]

## Data Availability

The raw RNA sequencing data have been submitted to the NCBI Short Read Archive (SRA) with accession number PRJNA1100473.

## Supplementary Materials

The following supporting information can be downloaded at: https://www.mdpi.com/article/10.3390/pathogens13070592/s1, Figure S1: Multiple sequence alignment of FgNhp6 with homologs from other fungi; Figure S2: Generation and identification of FgNhp6 deletion mutants; Table S1: All the identified differentially expressed genes between *Fusarium graminearum* PH-1 strain and ΔFgNhp6 mutant in transcriptomic analysis; Table S2: GO enrichment analysis of all the identified DEGs; Table S3: KEGG enrichment analysis of all the identified DEGs. Table S4: Primers used in this study.
