# Supplementary material for: The Non-Histone Protein FgNhp6 Is Involved in the Regulation of the Development, DON Biosynthesis, and Virulence of Fusarium graminearum
Source: Pathogens. 2024 Jul 16;13(7):592. doi: 10.3390/pathogens13070592 (PMC11279982; doi:10.3390/pathogens13070592)
Supplement: Supplementary file 1 [file pathogens-13-00592-s001.zip › Figures S1 and S2.pdf]

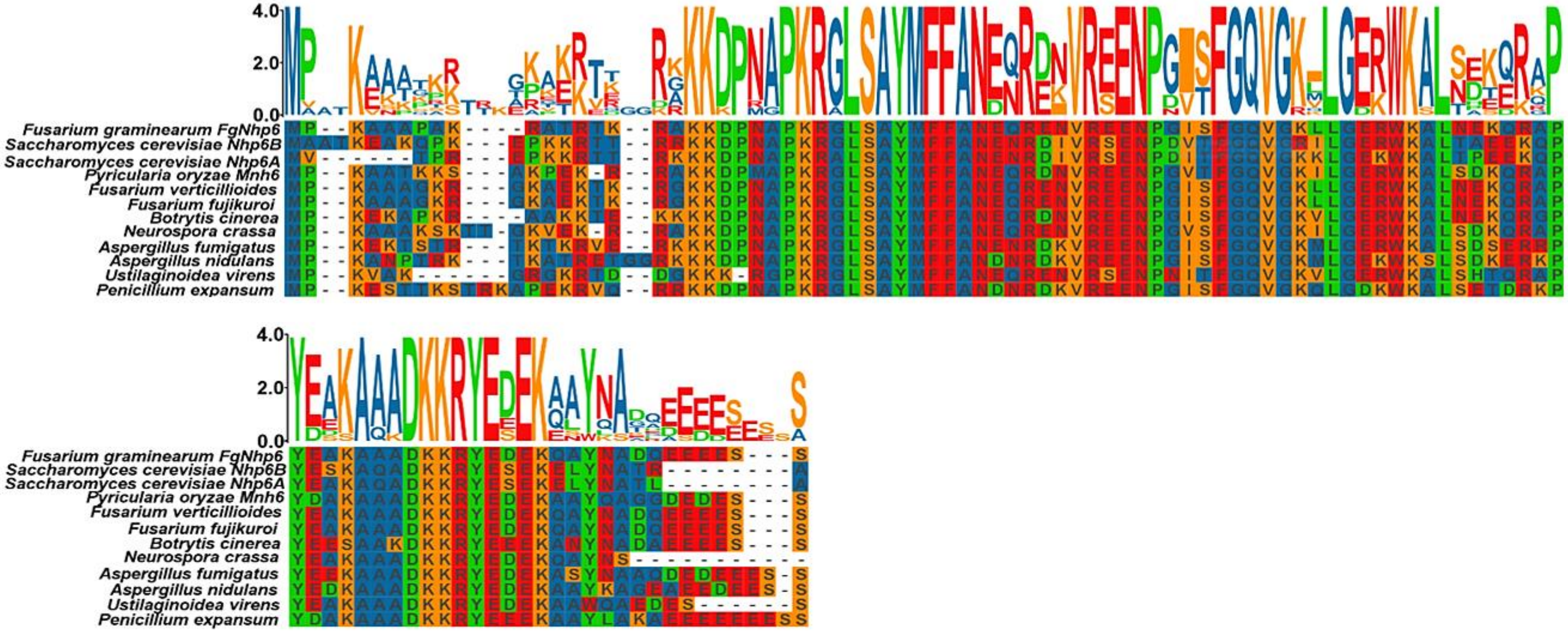

Figure S1. Multiple sequence alignment of FgNhp6 with homologs from other fungi. Sequence alignment was conducted using MAFFT. The sequence for analysis was as follows:

FgNhp6 from *Fusarium graminearum*, Nhp6B from *Saccharomyces cerevisiae*, Nhp6A from *Saccharomyces cerevisiae*, Mnh6 from *Pyricularia oryzae*, XP_018743258.1 from *Fusarium verticillioides*, XP_023424625.1 from *Fusarium fujikuroi*, XP_024551858.1 from *Botrytis cinerea*, XP_011393295.1 from *Neurospora crassa*, XP_754458.1 from *Aspergillus fumigatus*, XP_660489.1 from *Aspergillus nidulans*, XP_042995499.1 from *Ustilaginoidea virens*, XP_016594384.1 from *Penicillium expansum*.

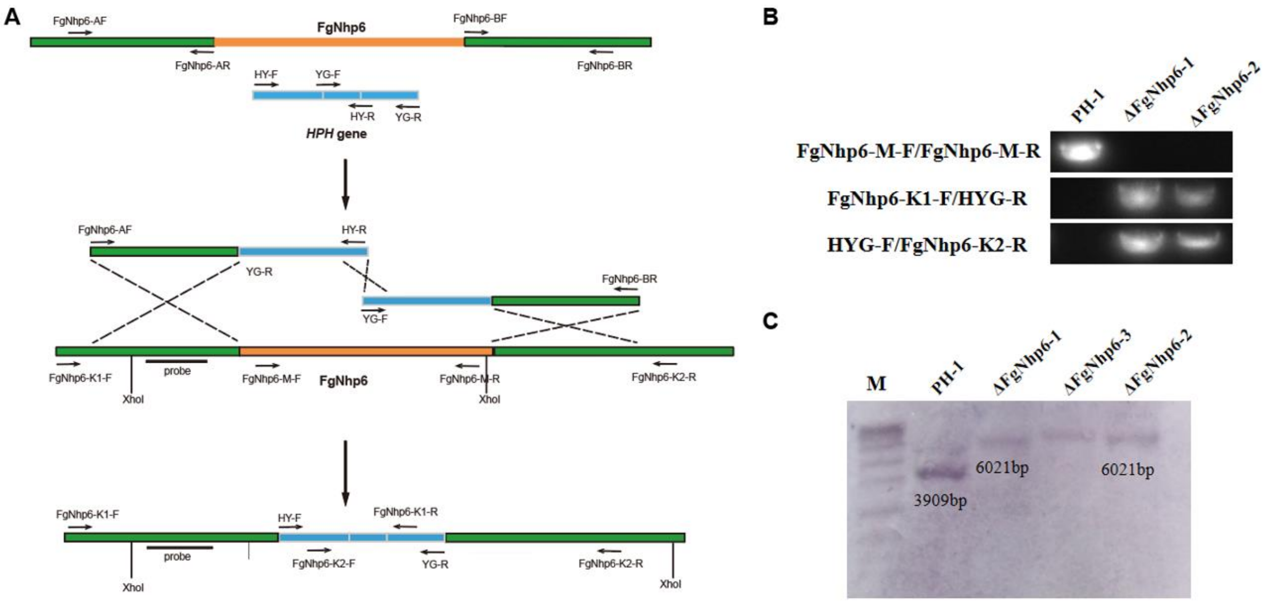

Figure S2. Generation and identification of FgNhp6 deletion mutants. (A) Schematic representation of the gene deletion strategy. The *FgNhp6* gene was replaced by the hygromycin gene (*HPH*) through homologous recombination. Primer binding sites are labeled by arrows. (B) PCR analysis of transformants showed ΔFgNhp6-1, -2 might be the correct FgNhp6 deletion mutants. (C) Southern blot analysis of FgNhp6 deletion mutants and the wild-type strain PH-1 confirmed the ΔFgNhp6-1 and ΔFgNhp6-2 were corrected. A 708 bp downstream fragment was used as a probe. The genomic DNA sample of each strain was digested with *Xho*I.
