# Supplementary material for: The Non-Histone Protein FgNhp6 Is Involved in the Regulation of the Development, DON Biosynthesis, and Virulence of Fusarium graminearum
Source: Pathogens. 2024 Jul 16;13(7):592. doi: 10.3390/pathogens13070592 (PMC11279982; doi:10.3390/pathogens13070592)
Supplement: Supplementary file 1 [file pathogens-13-00592-s001.zip › Table S4.pdf]

The non-histone protein FgNhp6 is involved in the regulation of the development, DON biosynthesis and virulence in *Fusarium graminearum*

Jiakuo Cao [1,†], Junbo Lv [1,†], Limin Zhang [1], Heng Li [1], Hao Ma [1], Yanxiang Zhao [1,*] and Jinguang Huang[1,*]

[1] College of Plant Health and Medicine, Shandong Engineering Research Center for Environment-Friendly Agricultural Pest Management, Qingdao Agricultural University, Qingdao 266109, P. R. China 910455209@qq.com (J.C.); jblyu@foxmail.com (J.L.); 2115753673@qq.com; 1477389576@qq.com (H.L.); 1727411440@qq.com (H.M.);

[*] Correspondence: zhaoyx@qau.edu.cn (Y.Z.); jghuang@qau.edu.cn (J.H.); Tel.: +86-532-58957365 (Y.Z.); +86-532-58957713 (J.H.)

[†] These authors contributed equally to this work.

Table S4 Primers used in this study.

Primer name	Sequence (5′-3′)	Application
FgNhp6-AF	ATGGACAAGCAGGGGATAAGA	To amplify the upstream fragment of *FgNhp6*
FgNhp6-AR	ttgacctccactagctccagccaagccAGCTCAAAGCGTGGACAGG	
FgNhp6-BF	gaatagagtagatgccgaccgcgggttCGATGAAAGTGGCTGATAGTGG	To amplify the downstream fragment of *FgNhp6*
FgNhp6-BR	TTAGGACGGGAATGAGTGGG	
HYG-F	GGCTTGGCTGGAGCTAGTGGAGGTCAA	To amplify the 5' terminal fragment (HY) of the hygromycin resistance gene *HPH*
HY-R	GTATTGACCGATTCCTTGCGGTCCGAA	
YG-F	GATGTAGGAGGGCGTGGATATGTCCT	To amplify the 3' terminal fragment (YG) of the hygromycin resistance gene *HPH*
HYG-R	AACCCGCGGTCGGCATCTACTCTATTC	
00385-K1-F	AATCCAACGCCCTTGAACA	For identification of FgNhp6 deletion transformants
00385-K2-R	CCTGTCGTCGCCTCAACTAA	
00385-M-F	AAAGCCCATCGCAAGTTCA	For identification of FgNhp6 deletion transformants
00385-M-R	ACACCGCCCAGACCAAAA	
00385-south-F	AGCGAGCAGAATGATGGGAGACGAC	To amplify the DNA fragment used as the probe for Southern blot analysis
00385-south-R	CGCTAACTATCCTACCTGTGCC	
ComNhp6-F	CCGGAATTCCTCTGGTGGAATGGGTTTGG	To amplify the DNA fragment used for the construction of the FgNhp6-complemented strain
ComNhp6-R	CGGGGTACCGGCGAGAATGACTGGGAAGA	
ComNhp6-GFP-F	CCGGAATTCCTCTGGTGGAATGGGTTTGG	To amplify the DNA fragment used for the construction of the FgNhp6-complemented strain
ComNhp6-GFP-R	CGGGGTACCGGCGAGAATGACTGGGAAGA	
Actin-RT-F	GAGAGCGGTCCTTCAATC	For qPCR analysis of *Actin* gene in *F. graminearum*
Actin-RT-R	TCCATGATCTTGTCGTTAGG	
AurF-RT-F	ATCTTCAGTCTTGACCATCCC	For qPCR analysis of *AurF* gene in *F. graminearum*
AurF-RT-R	TACCCAAGATGTTCTGGCAA	
AurJ-RT-F	GCCAGTTATCACACCATCTT	For qPCR analysis of *AurJ* gene in *F. graminearum*
AurJ-RT-R	GTTGCTTGTCATTGCCATAC	
PKS12-RT-F	CTGCTGTGTTAGTGATATGC	For qPCR analysis of *PKS12* gene in *F. graminearum*
PKS12-RT-R	GAGACGCTGGTTGCTATA	
GIP1-RT-F	TGCGGTATCAGGTCACAAA	For qPCR analysis of *GIP1* gene in *F. graminearum*
GIP1-RT-R	ATCAAAGTCTCCCACCGTGAA	
CYP51A-RT-F	CCGACATTACCGAAGAAC	For qPCR analysis of *FgCYP51A* gene in *F. graminearum*
CYP51A-RT-R	ATTGAGTGGATGGAAGAGT	
CYP51B-RT-F	TCCGTCGTCCTCAATGTC	

CYP51B-RT-R	CGTATGTGATGGTGCTTCC	For qPCR analysis of *FgCYP51B* gene in *F. graminearum*
CYP51C-RT-F	CTGAGTTAGAGCACGATGGAAT	For qPCR analysis of *FgCYP51C* gene in *F. graminearum*
CYP51C-RT-R	GACGATGGACAAGATGATGATGA	
ERG9-RT-F	TTACCAGCTTCGGTCCATTA	For qPCR analysis of *FgERG9* gene in *F. graminearum*
ERG9-RT-R	CACGGAGGCAGAGGTAGAAGA	
FGSG_03882-RT-F	GAACTCACGTACTCTCCTCAAC	For qPCR analysis of *FGSG_03882* gene in *F. graminearum*
FGSG_03882-RT-R	TCGACATCAACTCTCTCTTGAC	
FGSG_08308-RT-F	ACAGGCTCGTCTATTCACAG	For qPCR analysis of *FGSG_08308* gene in *F. graminearum*
FGSG_08308-RT-R	GACCCCTTGATAACCTCTAAC	
FGSG_08309-RT-F	GTCTCTTTGTATCAGGCTTCC	For qPCR analysis of *FGSG_08309* gene in *F. graminearum*
FGSG_08309-RT-R	CATATTCTCTCTCCCACTCATC	
FGSG_08749-RT-F	CGGTATTGGCGCTGGTTTCC	For qPCR analysis of *FGSG_08749* gene in *F. graminearum*
FGSG_08749-RT-R	CAGTGTCTAGAGCATCGAGAC	
FGSG_05096-RT-F	GACATGCATCGAGGCTCTAG	For qPCR analysis of *FGSG_05096* gene in *F. graminearum*
FGSG_05096-RT-R	GGAGCGGTAGTTTGGTAGGC	
FGSG_06871-RT-F	TCGTCTTGCCATATTCTTG	For qPCR analysis of *FGSG_06871* gene in *F. graminearum*
FGSG_06871-RT-R	TGAATGAGTGTCCTGTGA	
FGSG_06542-RT-F	GCCATTACTATACCGTCAAT	For qPCR analysis of *FGSG_06542* gene in *F. graminearum*
FGSG_06542-RT-R	CAAGAGTAGAACCAGGAGA	
FGSG_00574-RT-F	GAGGATGCCAACTTACTAC	For qPCR analysis of *FGSG_00574* gene in *F. graminearum*
FGSG_00574-RT-R	CCACAGTCCAGATAATTCG	
NoxA-RT-F	GAACTACAATCACGAACCA	For qPCR analysis of *FgNoxA* gene in *F. graminearum*
NoxA-RT-R	TTAATATCACGAGCAGCAG	
Pex1-RT-F	GCATCTATCTTCAGTTCTTCTC	For qPCR analysis of *FgPex1* gene in *F. graminearum*
Pex1-RT-R	GAGCGTATGGAACAATGATT	
Crz1A-RT-F	CTTCCAACATCAGTCCAA	For qPCR analysis of *FgCrz1A* gene in *F. graminearum*
Crz1A-RT-R	GAACATCTTATCGCAATCC	
Cdc15-RT-F	TGGCTAGGTGACGAAGAGGA	For qPCR analysis of *FgCdc15* gene in *F. graminearum*
Cdc15-RT-R	CGTGTCGATCTCGGGCAATA	
Chs1-RT-F	GTATATGCTTCTCACACCTA	For qPCR analysis of *FgChs1* gene in *F. graminearu*
Chs1-RT-R	GACTTCTCGTTGATATTGC	
Chs2-RT-F	GCCAACTTCTATCTCACTT	For qPCR analysis of *FgChs2* gene in *F. graminearum*
Chs2-RT-R	GGAAGATGACAGTAGCAATA	
ESA1-RT-F	TAAGCGACTGGATGAATG	

ESA1-RT-R	GTTGTGTCTTCTTGGATTG	For qPCR analysis of *FgESA1* gene in *F. graminearum*
SIRT2-RT-F	GAGTTCCTTCAGACAGAAT	For qPCR analysis of *FgSIRT2* gene in *F. graminearum*
SIRT2-RT-R	AGACAATATCAGGCTTGAC	
Tri4-RT-F	ATTCTATGGCAAGCACAA	For qPCR analysis of *TRI4* gene in *F. graminearum*
Tri4-RT-R	GCGGATACAAGAGTAAGG	
Tir5-RT-F	GATGGAGAACTGGATGGT	For qPCR analysis of *TRI5* gene in *F. graminearum*
Tri5-RT-R	TGCTTAGACGAGTGTAGG	
Tri6-RT-F	CTGTCGCTACTCAGAATG	For qPCR analysis of *TRI6* gene in *F. graminearum*
Tri6-RT-R	TTGTTGTCCTTCCTTGTC	
Tri10-RT-F	GCGACAGGAGCAAGAACATAA	For qPCR analysis of *TRI10* gene in *F. graminearum*
Tri10-RT-R	GGCGGCGTAAATCTGAGTG	
Tri11-RT-F	CTGTCGCTACTCAGAATG	For qPCR analysis of *TRI11* gene in *F. graminearum*
Tri11-RT-R	TTGTTGTCCTTCCTTGTC	
